# Sequelae associated with systemic hypertension in infants with severe bronchopulmonary dysplasia

**DOI:** 10.1038/s41372-022-01372-y

**Published:** 2022-03-30

**Authors:** Arvind Sehgal, Kristy Elsayed, Matilda Nugent, Suraj Varma

**Affiliations:** 1 Monash Newborn, Monash Children’s Hospital, Melbourne, Australia; 2 Department of Paediatrics, Monash University, Melbourne, Australia; 3 MonashHeart, Monash Health, Melbourne, Australia

**Keywords:** Respiratory tract diseases, Outcomes research

## Abstract

**Objectives:**

To ascertain correlation between systemic hypertension and respiratory sequelae amongst infants with BPD.

**Study design:**

Retrospective evaluation of six-year data compared infants with severe BPD to infants with no BPD. 7-day morning blood pressure (BP) (36^0^−36^6^ week) was compared with 95th centile cut-offs.

**Results:**

57 infants with BPD were compared with 114 infants with no BPD. Gestation and birthweight were comparable (median [interquartile range], (27 [25, 28] vs. 26.5 weeks [25, 28], *p* = 0.7 and 706 g [611, 884] vs. 730 [630, 895]), *p* = 0.1. Number of infants having BP ≥ 95th centile was significantly higher in BPD cohort (systolic BP, 23/57 [40.3%] vs. 3/114 [2.6%], *p* < 0.001 & mean arterial BP, 26/57 [46%] vs. 3/114 [2.6%], *p* < 0.001). Amongst BPD infants, higher BP was associated with longer duration of respiratory support (median [range], 109 days [81–138] vs. 87 [58–109], *p* < 0.001).

**Conclusions:**

Infants with severe BPD had higher BP compared to those without BPD.

## Introduction

Extremely premature infants are at risk of developing bronchopulmonary dysplasia (BPD), the most common respiratory sequelae of prematurity. Recent data from the Australian and New Zealand Neonatal Network (ANZNN) noted that the prevalence of BPD continues to be high (approximately 60%) in infants ≤ 26 weeks gestational age (GA) [1]. Approximately 40% of extremely low birthweight (BW) infants born in the United States develop BPD every year, affecting approximately 15,000 infants and putting immense burden on healthcare resources [2, 3]. The diagnostic and therapeutic approaches for BPD-associated *pulmonary* hypertension, and associated prognostic significance have been well-studied [4–6]. Similar information on *systemic* hypertension has not been characterized in recent neonatal literature. There is limited data regarding the occurrence of systemic hypertension and association with respiratory sequelae (duration of respiratory support) [7–9]. Higher blood pressure (BP) readings by a mean of 5 mm Hg have been noted in the neonatal period in infants with BPD compared to the non-BPD group [10]. These studies reflected a wide range in the occurrence of hypertension, had heterogeneity in the definition of BPD and used different cut-offs and timelines for BP recordings. More recent data also portrays BPD as a risk factor for systemic hypertension [11, 12]. The lack of robust reference BP values in infants contributes to the lack of consensus as to the most appropriate criteria for such cohorts. The 2017 clinical practice guideline for screening and management of high BP in children and adolescents from the American Academy of Pediatrics Clinical Practice Guideline Subcommittee on Screening and Management of High Blood Pressure in Children recommends the use of derived BP centiles based on post-menstrual age (PMA) [13]. Dionne and colleagues had earlier published estimated BP values in infants from 26 to 44 weeks postconceptional age [14]. These reference data provide for 95th and 99th centile readings, and are recommended for planning close monitoring, and initiating investigations and potential treatments depending on the clinical situation. The increased systemic arterial thickness and stiffness in infants with BPD [15], could plausibly increase afterload and may explain the left ventricular hypertrophy noted previously in similar infants [7]. In essence, the *systemic* constructs of severe BPD merit interrogation to understand pathophysiology and putative links with sequelae.

This study used the 95th and 99th centiles for BP according to the infant’s PMA [14]. The objectives of this study were to compare 7-day period (36^0^–36^6^ weeks postmenstrual age) BP amongst infants with severe BPD, to those with no BPD, and to ascertain correlation between systemic hypertension and respiratory sequelae amongst infants with severe BPD. Subsequently, clinical comparisons were made within the cohort of infants with BPD, between those with systolic BP ≥ 95th centile and < 95th centile. For the purpose of this study, hypertension was defined as systolic BP ≥ 95th centile for PMA.

## Methods

We performed a retrospective appraisal of archived clinical data for a six-year period (2014–2019). Demographic information was retrieved from medical records for preterm infants who survived to 36 weeks PMA. Infants who died before 36 weeks PMA or those with congenital heart disease (other than patent ductus arteriosus [PDA]) were excluded. These infants were born consecutively. In a 1:2 ratio, we compared infants with severe BPD to GA matched subsequent admissions who did not develop BPD. We reviewed demographic and clinical data including the use of umbilical artery catheters (UAC), PDA and medications such as postnatal steroids. For consistency, the daily first morning BP measurement was recorded for the 7-day period (36^0^ to 36^6^ weeks PMA). The primary reason to choose 36 weeks as the timeline was the ANZNN definition of BPD (requirement for respiratory support at 36 weeks PMA). This timeline would also obviate infants with transient hypertension due to factors such as initial use of inotropes or UAC. We used 95th centile cut-offs per PMA as a uniform timeline ensures a single cut-off to be used across the cohort. BP was measured in quiet state, between feeds as per standard nursing guidelines. The 95th and 99th centile for systolic BP were (87 and 92 mm Hg) and for mean arterial BP were (72 and 71 mm Hg) [12]. Additionally, we also recorded the 7-day average BP. BP recordings were made with appropriate size cuff using Drager Infinity M540 (Drägerwerk AG & Co. KGaA Moislinger Allee 53–55 23558 Lübeck, Germany). BPD severity was graded according to the National Institute of Health classification (severe BPD-need for ≥ 30% oxygen and/or positive pressure ventilation at 36 week PMA) [16]. For the purpose of the study, fetal growth restriction was defined as BW < 10th centile for GA and sex per locally used reference charts [17]. Antenatal Doppler recordings in the fetal arteries were either absent or reversed in diastole in all infants in the FGR cohort. The Institution Research Ethics Committee approved the study.

### Statistics

All analyses were performed using the Stata/BE 17 software (StataCorp, College Station, Texas, United States). We used general linear regression models to assess the effect of group (BPD and no BPD) on BP. To account for the confounding effects of variables such as growth restriction, surfactant administration, PDA, UAC, postnatal dexamethasone therapy and maternal characteristics (diabetes, pre-eclampsia and chorioamnionitis), adjusted analyses were performed by including these variables as covariates in the regression model. Continuous variables were summarized using means and standard deviations or median (range/interquartile range [IQR]), and analysed using *t*-test. Categorical variables were expressed as counts and proportions and were analysed using Chi-square or Fisher Exact Test as appropriate.

## Results

Fifty-seven infants with severe BPD and 114 preterm infants with no BPD formed the study cohort. All infants in the cohort were ≤ 30 weeks GA. The GA and BW between the groups were comparable (median [IQR], (27 [25,28] vs. 26.5 weeks [25,28], *p* = 0.7 and 706 g [611,884] vs. 730 [630,895]), *p* = 0.1, respectively. Maternal characteristics and the use of antenatal steroids, surfactant and UAC in the two groups were comparable (Table 1). The presence of PDA was significantly higher in the infants with BPD; almost all infants with PDA in the BPD cohort were medically treated 42/46 (91.3%). Diuretic use during 36^0^−36^6^ week was higher in infants with BPD; 11 (19%) vs 1 (0.9%), *p* < 0.001. The gestational timeline for the administration of postnatal steroids in the two groups was 30 ± 2 compared with 30 ± 1.4 weeks GA (*p* = 0.54). None of the infants were on inotropic support in the fortnight leading up to the BP assessments. Seven infants in the BPD group were administered captopril (GA at administration was 45.3 ± 6 weeks). Essentially, no infant was on anti-hypertensives during/leading up to the week of assessments. No infant in the non-BPD group was administered anti-hypertensives.

**Table 1:** Demographic and clinical data of the study population.

Variable	Preterm BPD (*n* = 57)	Preterm no BPD (*n* = 114)	*P*
Gestational age (weeks)*	27 (25, 28)	26.5 (25, 28)	0.7
Birthweight (g)*	706 (611, 884)	730 (630, 895)	0.1
Antenatal steroids	55 (96.5)	106 (93)	0.5
Fetal growth restriction	21 (37)	5 (4.4)	<0.001
Male sex	32 (56)	69 (60.5)	0.6
5 min Apgar score^	7 (0–10)	7 (1–9)	0.6
Umbilical artery catheter	35 (56)	52 (45.6)	0.07
Surfactant	51 (89.5)	100 (87.7)	0.8
PDA	46 (81)	28 (24.5)	<0.001
PDA therapy amongst those diagnosed with PDA	42 (91.3)	20 (71.4)	0.047
Postnatal steroids	32 (56%)	17 (15%)	<0.001
Maternal diabetes	10 (8.7%)	4 (7%)	0.7
Pre-eclampsia	18 (16%)	6 (10.5%)	0.5
Chorioamnionitis	12 (10.5%)	7 (12%)	0.8
Duration of any respiratory support, days, median (range)	96 (58–138)	44 (10–89)	<0.001

Data presented as ^*^median (interquartile range), ^^^median (range). Rest all data in *n* (%),

*PDA* Patent ductus arteriosus, *BPD* Bronchopulmonary dysplasia.

**Fig. 1:**
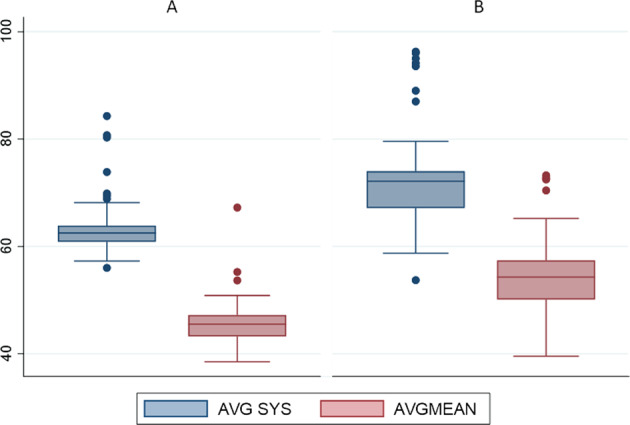
7-day average blood pressure (BP) data for the study population in mmHg. **A** Systolic and mean arterial BP in infants with no bronchopulmonary dysplasia (BPD). **B** Systolic and mean arterial BP in infants with BPD. Boxplot displays the ‘minimum (Q1 −1.5*IQR)’, first quartile (Q1), median, third quartile (Q3), and ‘maximum (Q3 + 1.5*IQR)’ with outliers. IQR Interquartile range.

The number of infants having multiple readings ≥ 95th centile during the 7-day period between 36^0^−36^6^ weeks PMA was significantly higher in the BPD cohort compared to infants with no BPD (systolic BP, 23/57 [40.3%] vs 3/114 [2.6%], *p* < 0.001 & mean arterial BP, 26/57 [46%] vs. 3/114 [2.6%], *p* < 0.001). Six BPD infants (10.5%) had 7-day systolic BP average ≥ 95th centile while nine (15.8%) had 7-day mean arterial BP average ≥ 95th centile. Table 2 compares systolic, diastolic, and mean arterial BP during the study period. Five (9%) infants in the BPD group had 7-day average systolic BP ≥ 99th centile (none in the no BPD group). The unadjusted 7-day average systolic and mean arterial BP was significantly higher in infants with BPD compared to infants with no BPD (median [range], 72 mm Hg [54–96], {95% confidence interval 70, 75} vs. 63 [56–84], {95% confidence interval 62, 64}, *p* < 0.001 and 54 mm Hg [40–73], {95% confidence interval 53, 56} vs. 45 mm Hg [39–67], {95% confidence interval 44, 47}, *p* < 0.001), respectively. The same data for diastolic BP was 46 mm Hg (32–62), {95% confidence interval 44, 48} vs. 37 mm Hg (28–59), {95% confidence interval 36, 38}, *p* < 0.001. Figure 1 depicts the above data for the two groups as box plots. Table 3 depicts logistic regression analysis using maternal and infant variables. Table 4 compares infants with BPD who had systolic BP ≥ 95th centile with those < 95th centile. The GA, BW and other common neonatal variables between the two cohorts were comparable. While higher BP was associated with greater odds (3.5 [95% confidence interval, 0.7, 16] for discharge with home oxygen, this was statistically not significant. The duration of respiratory support includes any support, including continuous positive airway pressure or low flow. The timeline includes the whole hospitalisation (and duration of home oxygen [for those discharged in home oxygen]). Seven of 23 (30.4%) infants with BPD with hypertension were administered captopril (none in the no BPD group), for a variable period ranging from 3 to 6 months. Clinical diagnosis of hypertension was recorded for these seven infants. No infant in the study had coarctation of aorta. Renal ultrasound was done in 46 infants with BPD; no evidence of renal artery stenosis or multicystic dysplastic kidney was noted.

**Table 2:** Systolic and mean arterial blood pressure data of the cohort (36^0^−36^6^ weeks post-menstrual age).

Gestational age (weeks)	BPD-SBP *n* = 57	No BPD-SBP *n* = 114	*p*-value
36^0^			
SBP	70 (44–100)	61 (50–88)	< 0.001
MBP	50 (34–77)	46 (37–68)	< 0.001
DBP	42 (22–66)	38 (28–58)	0.002
36^1^			
SBP	70 (50–101)	62 (50–91)	< 0.001
MBP	53 (35–77)	45 (38–69)	< 0.001
DBP	44 (25–66)	36 (28–58)	< 0.001
36^2^			
SBP	70 (49–99)	61 (43–88)	< 0.001
MBP	56 (36–77)	44 (37–69)	< 0.001
DBP	47 (30–66)	35 (28–60)	< 0.001
36^3^			
SBP	70 (41–100)	62 (48–86)	< 0.001
MBP	54 (30–85)	45 (35–69)	< 0.001
DBP	47 (24–78)	36 (28–61)	< 0.001
36^4^			
SBP	71 (50–100)	64 (52–82)	< 0.001
MBP	57 (33–77)	44 (37–66)	< 0.001
DBP	48 (25–66)	35 (28–58)	< 0.001
36^5^			
SBP	70 (54–98)	64 (50–84)	< 0.001
MBP	55 (39–73)	45 (38–64)	< 0.001
DBP	47 (30–60)	35 (28–58)	< 0.001
36^6^			
SBP	70 (58–96)	64 (55–81)	< 0.001
MBP	55 (41–73)	45 (38–66)	< 0.001
DBP	47 (31–65)	36 (28–58)	< 0.001

*SBP* Systolic blood pressure, *MBP* Mean arterial blood pressure, *DBP* Diastolic blood pressure, *BPD* Bronchopulmonary dysplasia,

*BP* Data in mm Hg, presented as median (range).

**Table 3:** Regression analysis for variables which might influence 7-day average systolic blood pressure (AVGSYS).

AVGSYS	Coefficient	Standard error	*t*	*P* > ǀ t ǀ	95% confidence interval
BPD	9.36	1.46	6.42	0.000	6.5–12.2
FGR	−0.58	2.33	−0.25	0.8	−5.2–4
Surfactant	−0.08	1.62	−0.05	0.96	−3.3–3.1
PDA	1.7	1.31	1.29	0.19	−0.9–4.3
POSTNATALDEXYN	−1.93	1.37	−1.41	0.16	−4.6–0.8
UAC	−1.31	1.14	−1.15	0.25	−3.6–0.9
MatrDM	0.9	1.9	0.47	0.63	−2.8–4.6
PREECL	1.7	2.2	0.77	0.44	−2.6–6
CHORIO	0.44	1.71	0.26	0.8	−2.9–3.8
_cons	63.4	1.52	41.6	0.000	60.4–66.4

*BPD* Bronchopulmonary dysplasia, *FGR* Fetal growth restriction, *PDA* Patent ductus arteriosus, *POSTNATALDEXYN* Postnatal dexamethasone,

*UAC* Umbilical artery catheter, *MatrDM* Maternal diabetes mellitus, *PREECL* Pre-eclampsia, *CHORIO* Chorioamnionitis.

R-squared = 0.37, number of observations = 171.

**Table 4:** Comparison of infants with SBP ≥ or < 95th centile, within the BPD cohort.

Variable	Infants with SBP ≥ 95 centile, *n* = 23	Infants with SBP < 95 centile, *n* = 34	*P* value
Gestational age (weeks)*	26 (25, 27)	27 (25, 28)	0.049
Birthweight (g)*	786 (710, 990)	700 (596, 858)	0.28
FGR	6 (26)	15 (44)	0.26
Umbilical artery catheter	11 (48)	24 (70)	0.1
Surfactant replacement therapy	21 (91)	30 (88)	1
PDA	19 (83)	27 (79)	1
PDA therapy	18 (78)	24 (70)	0.55
Use of postnatal steroids	14 (61)	18 (53)	0.6
Maternal diabetes	4 (17)	6 (17)	1
Pre-eclampsia	8 (35)	10 (29)	0.8
Chorioamnionitis	5 (22)	7 (20)	0.2
Duration of any respiratory support days, median (range)	109 (81–138)	87 (58–109)	< 0.001

Data presented as ^*^median (interquartile range) or *n* (%), *FGR* Fetal growth restriction, *PDA* Patent ductus arteriosus, *SBP* Systolic blood pressure, *BPD* Bronchopulmonary dysplasia.

## Discussion

The assessment, monitoring and treatment of BPD associated pulmonary hypertension has dominated BPD literature to the extent of relegating the systemic constructs of this complex disease to a footnote. The contribution of left-sided (systemic) circulation towards BPD pathophysiology is not particularly appreciated. Systemic hypertension seems to be a significant issue in BPD infants, with important implications for management as well as prognosis.

### Information from previous literature

Abman and colleagues first indicated the presence of systemic hypertension in infants with BPD [7]. In this study, BPD was defined based on the need for positive pressure ventilation in the first week of life, clinical respiratory distress and oxygen dependency persisting beyond one month of age, and radiographic evidence of BPD. The diagnosis of hypertension was made if the systolic BP was >133 mm Hg on at least three separate occasions, based on the criteria of de Swiet [18]. In this retrospective study, 13/30 (43%) infants with BPD demonstrated hypertension. The mean age of onset ranged from 0.5 to 15 months and more than half were diagnosed after neonatal intensive care discharge. Six of 13 (46%) infants were treated with antihypertensive medications (propranolol and hydralazine). Only one of the 22 infants without BPD developed hypertension. Anderson et al retrospectively studied 87 infants with BPD using the above criteria for BPD and hypertension, and noted that it was present in 11/87 (13%) infants, mean age of onset being 6 months (range, 1.2–10.3 months) [8]. Two of these 11 infants (18%) were treated with captopril. Alagappan and colleagues retrospectively studied 73 infants, which included 41 infants with BPD (using the above-mentioned definition for BPD). The BP data was gathered from archived records on day 7, 28, 42 and around the time of discharge, and hypertension was defined as mean arterial BP ≥ 105 mm Hg on any one of those days [9]. Five of 41 (12%) infants with BPD had hypertension; only one of these five (20%) were treated using hydralazine. A recent retrospective study defined systemic hypertension when 3 separate measurements of systolic BP were > 95th centile [12]. Over a 4-year period, 53 (1.3%) infants had hypertension; of whom 74% were preterm. BPD was identified as a major risk factor.

Our study differs from previous literature in many ways. Salient amongst them include the use of a contemporary definition of BPD (respiratory support dependence at 36 weeks PMA), the use of recent 95th and 99th centile for cut-offs, and comparison of infants with BPD with equally preterm infants with no BPD. Our study on 171 preterm infants noted significantly higher BP readings amongst those with BPD, compared to equally premature infants with no BPD. In addition, higher BP was associated with a longer duration of respiratory support requirements and discharge on home oxygen.

### Possible associations underlying systemic hypertension

Reported risk factors for the development of systemic hypertension amongst neonates include UAC and the use of postnatal corticosteroids for lung disease [19–21]. Recent data has not been able to find any significant differences regarding the use of UAC in infants with BPD with or without hypertension [7–9, 12]. Our results mirror these findings as we noted that the presence of UAC was comparable between infants with or without BPD as well as among infants with BPD-with or without hypertension. A recent systematic review and meta-analysis assessed the role of postnatal corticosteroids for the prevention of BPD. A medium cumulative dose (2–4 mg/kg) of systemic dexamethasone was associated with higher risk of hypertension (defined in that study as systolic or diastolic BP two standard deviation above the mean for neonates’ GA and postnatal age) (grade of recommendation: low) [21]. While the use of postnatal steroids amongst the infants with BPD in our study was higher, an expected finding, there was no significant difference between those with systolic BP ≥ 95th centile and <95th centile within the BPD cohort. Similar comparable use of postnatal steroids between hypertensive and non-hypertensive infants with BPD has been reported earlier [8, 9].

There is a higher proportion of FGR in the infants with BPD, although the proportions within the BPD cohort are comparable between those with and with no hypertension. FGR as a risk factor for BPD as well as hypertension is known. A multicentre retrospective study noted that, at 27 weeks GA, approximately 25% of infants without FGR developed BPD compared to 60% with moderate FGR and 90% with severe FGR [22]. Other contemporary and recent studies on preterm infants also identified similar associations [23–25]. Our group recently highlighted FGR and hypertension in the offspring from the perspectives of mechanistic linkage and therapeutic directions [26].

### Mechanisms underlying higher BP in infants with BPD

Higher BP readings in infants with severe BPD could have multiple underlying explanations. Hypoxia and hypercarbia increase systemic vascular resistance through stimulation of peripheral arterial chemoreceptors. The latter could cause catecholamine release and increased vasomotor tone [27]. Decreased pulmonary vascular clearance or even net production of circulating catecholamines has previously been noted in infants with BPD [28, 29]. Inflammation causes abnormal collagen deposition and endothelial dysfunction via multiple pro-inflammatory cytokines, complementing the role of angiotensin II [30–32].

### Clinical relevance and pathophysiology driven therapeutic pathways

It is tempting to draw parallels between the prognostic role of arterial stiffness and hypertension in the systemic circulation, to pulmonary arterial stiffness and its prognostic value in pulmonary hypertension [33, 34]. Longer duration of hospital stay, home oxygen support and left ventricular hypertrophy have been previously noted in hypertensive BPD infants compared to the normotensive group [7–9]. Results of our study mirror findings from previous literature where the duration of respiratory support requirement was significantly higher in infants with BPD with elevated BP. In a series of observational and prospective echocardiographic studies, we have demonstrated the relevance of systemic hemodynamics in BPD pathophysiology and clinical outlook [30, 35, 36]. In a cohort of preterm infants with severe BPD, compared to preterm infants with no BPD and healthy term infants, cardiac indices reflecting systemic afterload as pathophysiologic factors were assessed [30]. Hypertension and systemic artery driven afterload may contribute to pathophysiology and sequelae, and preliminary data noting respiratory and cardiac function improvements with afterload reduction [30, 37], suggest biological plausibility that addressing the systemic constructs of BPD maybe effective in a subset of infants with severe BPD, and merits further exploration. Amongst infants with BPD, the cohort with severe BPD are on higher respiratory support, have significantly poorer respiratory outlook and prognosis, and have been the focus of much pulmonary research. Hence our cohort selection of infants with severe BPD. Nonetheless, a broader cohort of all BPD (mild to severe) would be more informative in future studies.

The limitation of relatively small numbers is accepted, although the overall cohort is larger compared to pre-existing literature. The Unit has a practice of measuring BP during quiet state and between feeds, though the retrospective nature of data collection through archived medical records is accepted as a limitation in this regard. Renin levels were not routinely collected and we did not assess placental histopathology of the study cohort. We limited our data collection to the 7-day period from 36^0^ to 36^6^ weeks, to coincide with the ANZNN definition of BPD. As previous data has shown, more than half of the infants with elevated BP are noticed post-neonatal intensive care discharge during follow-up visits. In that respect, our data could be under-estimating the burden of disease. On the other hand, some infants might have transient hypertension and may represent an over-estimation. We submit that a longitudinal follow-up study comparing infants with BPD and no BPD is urgently required. Systematic collection of BP data by neonatal networks would further detail the burden of disease. Large-scale population based data may help refine BP thresholds and guide choice of anti-hypertensive therapy.

## Conclusions

Our study found a higher proportion of infants with severe BPD had hypertension. Presence of hypertension in infants with severe BPD correlated with adverse respiratory outlook.

## Data Availability

The datasets generated during and/or analysed during the current study are available from the corresponding author on reasonable request.
